# Macrophages in inflammation and its resolution

**DOI:** 10.3389/fimmu.2012.00324

**Published:** 2012-11-01

**Authors:** Amiram Ariel, Isabelle Maridonneau-Parini, Patrizia Rovere-Querini, Jerrold S. Levine, Heiko Mühl

**Affiliations:** ^1^University of HaifaHaifa, Israel; ^2^CNRS, UMR5089, IPBS (Institut de Pharmacologie et de Biologie Structurale)Toulouse, France; ^3^Université de Toulouse, UPS, IPBSToulouse, France; ^4^San Raffaele Scientific Institute MilanoMilano, Italy; ^5^University of Illinois at ChicagoIL, USA; ^6^University Hospital Goethe UniversityFrankfurt am Main, Germany

Macrophages are highly plastic leukocytes that differentiate from monocytes following their entry into extravascular tissues. Macrophages can enter various tissues under inflammatory or non-inflammatory conditions and assume different functions and phenotypes according to the cues they receive from the environment. The notion that inflammation in general and macrophage responses in particular affect physiological phenomena that were previously considered to be not immune-related has enhanced and broadened our understanding of macrophage function during inflammation and its resolution.

This volume brings together 14 manuscripts that cover various aspects of macrophage function during inflammation and its resolution, as well as in several pathologic states for which a significant, long-lasting, macrophage-mediated immune response plays a significant role. Two of the manuscripts present original research on macrophage phagocytosis and its implications. Five provide an overview of macrophage function during inflammation and its resolution, with an emphasis on the modulatory role of particular elements in this response, such as apoptotic leukocytes, specific pathogens, hypoxia, and hormone receptors. The remaining seven manuscripts outline the role of macrophages during inflammation and its resolution in different tissues, including the lung, cardiovascular and adipose tissues, injured skeletal muscle and neuronal tissues, and synovial and oral cavities.

The two original research articles are devoted to the consequences of particle engulfment by macrophages. Labrousse et al. (2011) describe a novel experimental strategy in which they use micro-patterned immune complexes to trigger frustrated phagocytosis and thereby determine spatial parameters in lysosomal movement and fusion. Janko et al. (2011) report on the cumulative binding of CRP and anti-CRP antibodies to the surface of secondary necrotic cells. This binding leads to a pro-inflammatory cytokine response following engulfment by macrophages, implying a potential role for these elements in the etiology of systemic lupus erythematosus.

Of the review articles that discuss the regulation of macrophage differentiation and function by discrete events, two cover the interaction between macrophages and apoptotic leukocytes during the resolution of inflammation. Korns et al. (2011) outline the regulation of apoptotic cell clearance by macrophages (efferocytosis) and the environmental cues that promote the efferocytic capabilities of macrophages. The second manuscript by Ariel and Serhan (2012) reviews the impact of apoptotic cell sensing and disposal by macrophages on the switches in functional phenotypes displayed by these cells. The effect of another environmental factor, hypoxia, on monocyte/macrophage activation, and differentiation through transcriptional and translational modulation is covered by Rahat et al. (2011). Lugo-Villarino et al. (2011) discuss the pathogenesis and co-mortality displayed by two macrophage-inhabiting microbes (HIV and Mycobacterium Tuberculosis) and their influence on macrophage polarization. Patel et al. (2011) review the role of melanocortin receptor expression by macrophages in anti-inflammation and the resolution of inflammation, with attention given to melanocortin receptor agonists as therapeutic agents.

Several review articles discuss the function of macrophages during inflammation and/or its resolution within distinct anatomical sites, taking into account the unique features of these tissue-specific macrophages, in particular the distinct environments in which they reside and their interactions with neighboring cells. Clària et al. (2011) review current knowledge on the contribution of macrophages to the inflammatory state characterizing adipose tissue and the phenotypic changes observed in macrophages during obesity. Kennedy et al. (2011) discuss macrophage polarization occurring within the synovial space of arthritic joints and its modulation by cytokines, transcription factors, and pro-resolving lipid mediators. The article from Bosurgi et al. (2011) describes the multiple actions of macrophages in injured skeletal muscle, where the effects of these cells are a double-edged sword and can either promote healing and repair or lead to fibrosis and fat replacement. Herold et al. (2011) survey the indispensable role of macrophages in the resolution and termination of inflammation in lung infection and injury as well as the molecular pathways involved in these processes. Proper termination of inflammatory events and clearance of apoptotic cells are also critical to the cardiovascular system, as reviewed by Thorp (2012), and defects in macrophage efferocytosis can lead to atherosclerosis and myocardial infarction. While monocyte-derived macrophages and resident microglia were previously considered to be detrimental in brain inflammation and injury, recent advances reviewed by Jung and Schwartz (2012) suggest an opposite role for these macrophage-like cells, with a positive impact on brain maintenance and repair. Finally, Hasturk et al. (2012) outline the reciprocal interaction between periodontal disease and chronic inflammatory illnesses and the role that macrophages play in mediating these chronic inflammatory diseases.

Altogether, the articles in this volume portray the complexity of the multiple roles played by macrophages and members of their lineage during inflammation and its resolution, and their manipulation by the injured milieu. These topics are currently heavily studied, and advances in the field, facilitated by state-of-the-art genetics and optical technologies, will undoubtedly continue to contribute to our understanding of the immune system's response to foreign insults, trauma, and inflammatory disorders.
